# Validation and Evaluation of Reference Genes for Quantitative Real-Time PCR in *Macrobrachium Nipponense*

**DOI:** 10.3390/ijms19082258

**Published:** 2018-08-01

**Authors:** Yuning Hu, Hongtuo Fu, Hui Qiao, Shengming Sun, Wenyi Zhang, Shubo Jin, Sufei Jiang, Yongsheng Gong, Yiwei Xiong, Yan Wu

**Affiliations:** 1 Wuxi Fishery College, Nanjing Agricultural University, Wuxi 214081, China; huxiaoao@163.com; 2 Key Laboratory of Freshwater Fisheries and Germplasm Resources Utilization, Ministry of Agriculture, Freshwater Fisheries Research Center, Chinese Academy of Fishery Sciences, Wuxi 214081, China; qiaoh@ffrc.cn (H.Q.); sunsm@ffrc.cn (S.S.); zhangwy@ffrc.cn (W.Z.); jinsb@ffrc.cn (S.J.); jiangsf@ffrc.cn (S.J.); gongys@ffrc.cn (Y.G.); xiongyw@ffrc.cn (Y.X.); wuy@ffrc.cn (Y.W.)

**Keywords:** *Macrobrachium nipponense*, reference gene, normalization, quantitative real-time PCR

## Abstract

Quantitative real-time PCR (qPCR) is widely used in molecular biology, although the accuracy of the quantitative results is determined by the stability of the reference genes used. Recent studies have investigated suitable reference genes for some crustaceans under various conditions, but studies in *Macrobrachium nipponense* are currently lacking. In this study, we selected the following seven genes from among 35 commonly used housekeeping genes as candidate qPCR reference genes for temporal and spatial expression: *EIF* (*eukaryotic translation initiation factor 5A*), *18S* (*18S ribosomal RNA*), *EF-1α* (*elongation factor-1α*), *GAPDH* (*glyceraldehyde-3-phosphate dehydrogenase*), *TUB* (*α-tubulin*), *β-act* (*β-actin*), and *RPL18* (*Ribosomal protein L18*). The stability of each reference gene was evaluated by GeNorm, NormFinder, BestKeeper, and comparative ∆C _t_ methods, and was comprehensively ranked using RefFinder. *RPL18* was shown to be the most suitable reference gene for adult *M. nipponense* tissues, while *EIF* was the most stable in different ovarian and embryo stages and in white spot syndrome virus infection, and *β-act* was the most stable reference gene under hypoxia stress. The reliability of the rankings was confirmed by RNA interference experiments. To the best of our knowledge, this represents the first systematic analysis of reference genes for qPCR experiments in *M. nipponense*, and the results will provide invaluable information for future research in closely related crustaceans.

## 1. Introduction

Quantitative real-time PCR (qPCR) is a widely used technique for investigating gene expression levels, with high accuracy and sensitivity, as well as a wide application. Real-time PCR can involve relative or absolute quantification methods, of which the relative quantification is simple and accurate [1,2]. Housekeeping genes (HKGs), as known as reference genes [3], are used to account for alignment errors (e.g., as a result of the differences in RNA concentration, efficiencies, and reverse transcription), thus allowing the expression levels of the target gene to be calculated relative to the housekeeping gene [4,5,6]. However, the appropriate reference genes need to be searched and used for specific experimental conditions, because there is no perfect reference gene that maintains a stable expression in all of the tissues and conditions [7,8]. Furthermore, the use of inappropriate reference genes may result in conflicting gene expression data for different tissues or situations [9,10].

Molecular research in crustacean has become popular in recent years, and numerous studies have examined the growth, development, reproduction, and sex differentiation of crustaceans [11]. qPCR has been widely used in these studies, and some research has been carried out to screen and identify suitable reference genes in crustaceans. One study showed that the commonly used reference gene *β-act* was not the most stable reference gene in *Penaeus stylirostris* infected with white spot syndrome virus (*WSSV*) [12]. In *Peneaus monodon*, the appropriate reference gene for the reproductive gene expression profile was identified [13]. Furthermore, the reference genes were screened before starting a quantitative study of heat-shock responses in *Palaemonetes varians* [14], while studies in *Macrobrachium rosenbergii* and *Macrobrachium olfersii* showed that reference genes were not universal, and the most appropriate reference gene depended on the specific conditions [15,16].

The oriental river prawn *Macrobrachium nipponense* is widely distributed in freshwater and low-salinity estuarine regions in China, and is of great market value. Although many molecular studies have investigated the reproduction, sexual control, stress, and nutrition in *M. nipponense* [17,18,19], most qPCR studies were performed using reference genes from other similar species without identification and verification, potentially leading to inaccurate results. It is therefore essential to screen for suitable specific reference genes in *M. nipponense* under different experimental conditions.

In the current study, we investigated several reference genes based on other model animals and crustaceans. After comparing them with the *M. nipponense* transcriptome data and gel electrophoresis detection, we identified seven candidate reference genes in the *M. nipponense* transcriptome library. We then measured the expression stability of those genes in different adult tissues, ovarian and embryo stages, under hypoxia stress, and in white spot syndrome virus (*WSSV*) infection, and analyzed the results using GeNorm [20], NormFinder [20], BestKeeper [21], and the comparative ∆C_t_ method [22], and ranked them using the web-based comprehensive tool RefFinder [23]. We also performed RNA interference (RNAi) experiments to verify the accuracy of the screened reference genes by detecting the expression of *SST* (*slow-tonic S2 tropomyosin*) gene before and after the RNAi, using difference reference genes for normalization. This study represents the first comprehensive systematic screening of reference genes for *M. nipponense* based on experiments involving temporal and spatial expression and stress. Furthermore, it also provides the first results of the reference gene screening and the verification of reference genes by RNAi experiments in crustaceans. The results of this study fill a gap in the *M. nipponense*-related research, thus increasing the accuracy and reliability of future research into the expression of target genes, and providing a useful reference for studies in other crustaceans.

## 2. Results

### 2.1. Selection of Target Internal Reference Genes

Based on previous research in model organisms (*Danio rerio, Mice, Bactrocera dorsalis*, and *Oryza sativa*) and crustaceans (*Procambarus clarkia, Macrobrachium rosenbergii*, and *Macrophthalmus japonicas*), we screened 35 candidate genes in the transcriptome libraries of *M. nipponense* [24,25,26,27]. Fifteen of the genes were ubiquitously expressed in different tissue libraries and passed the BLAST test. Further screening identified seven reference genes with effective specificity and amplification, *EIF* (*eukaryotic translation initiation factor 5A*), *18S* (*18S ribosomal RNA*), *EF-1α* (*elongation factor-1α*), *GAPDH* (*glyceraldehyde-3-phosphate dehydrogenase*), *TUB* (*α-tubulin*), *β-act* (*β-actin*), and *RPL18* (*Ribosomal protein L18*). The open reading frames of these sequences were verified using the primers in Table A1. The sequences of the candidate genes were submitted to NCBI GenBank (Table A1).

### 2.2. Primer Specificity and Amplification Efficiency for qPCR 

**Figure 1:**
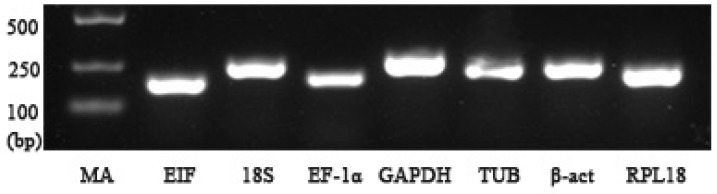
Agarose gel electrophoresis of qPCR primers amplification of the candidate reference genes using hepatopancreas cDNA as a template. MA means DNA marker DL2000, which is in the left side and shows their expected sizes. *EIF*—eukaryotic translation initiation factor 5A; *18S*—*18S ribosomal RNA*; *EF-1α*—*elongation factor-1α*; *GAPDH*—*glyceraldehyde-3-phosphate dehydrogenase*; *TUB*—*α-tubulin*; *β-act*—*β-actin*; *RPL18*—*Ribosomal protein L18*.

**Figure 2:**
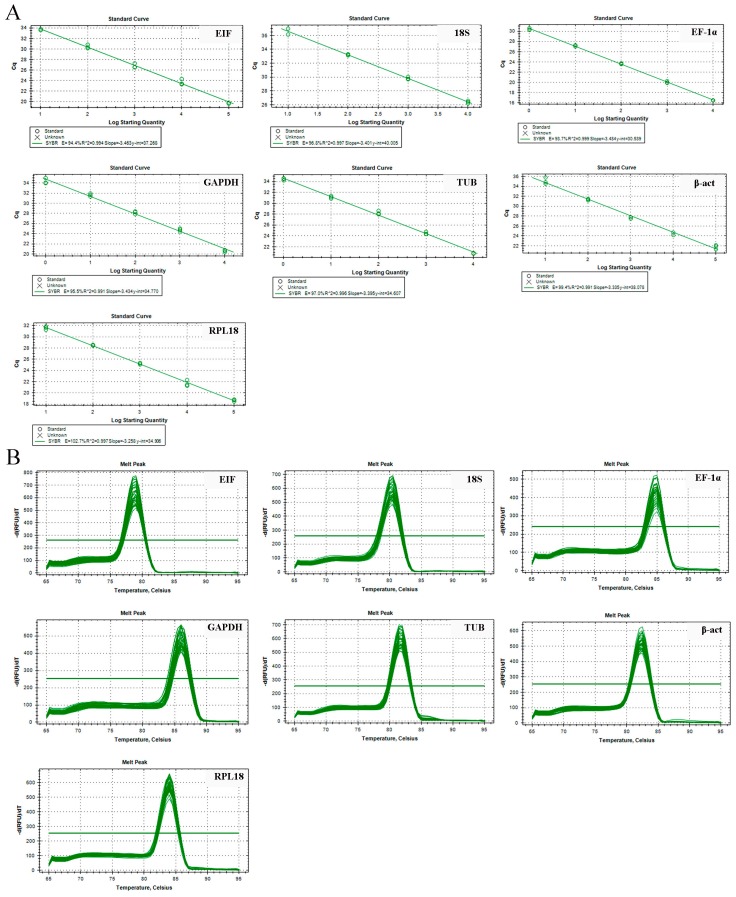
Dissolution and standard curve to verify the gene amplification efficiency and uniformity. (**A**) Dissolution curve of candidate reference genes. (**B**) Standard curve of candidate reference genes.

The qPCR primer pairs of each reference gene are presented in Table 1. The specificity of each single PCR product was confirmed by 1.2% agarose gel electrophoresis (Figure 1), and was matched with their sizes. The amplification efficiencies of these primers ranged from 0.93–1.02 (Table 1) and the standard curve for each gene from the cDNA dilutions displayed R^2^ > 0.99 (Figure 2A); each primer produced a single melting peak (Figure 2B), reflecting their stability and specificity [3].

### 2.3. Distribution of Cycle Threshold (Cq) Values 

The detailed Cq values are presented in Table 2. The Cq values of these candidates’ reference genes ranged from 17.75 (*β-act*) and 37.48 (*EF-1α*). A high Cq value represented a low expression level [3], indicating that in seven genes, *EIF, β-act*, and *RPL18* had the highest expression levels, and *EF-1α* and *18S* had the lowest levels.

**Figure 3:**
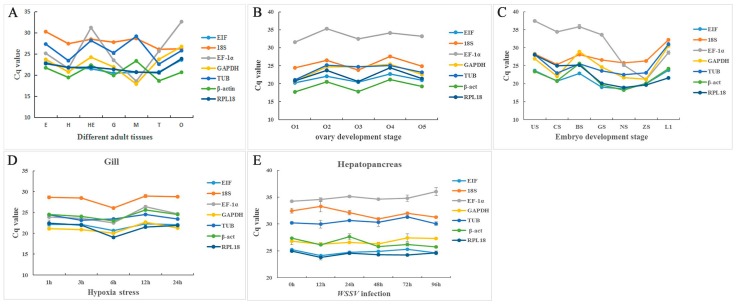
Variation in the reference genes expression using distribution of cycle threshold (Cq) values in line charts. (**A**) Different tissues in adult shrimps. (**B**) Five different ovary development stages, O1–5: Stage I, Stage II, Stage III, Stage IV, and Stage V of ovary. (**C**) Different embryo development stages, US—unfertilized egg stage; CS—cleavage stage; BS—blastula stage; GS—gastrul stage; NS—nauplius stage; ZS—zoea stage; L1—one day after larvae hatched. (**D**) Different times after hypoxia stress in gill. (**E**) Different times after infection in hepatopancreas.

**Figure 4:**
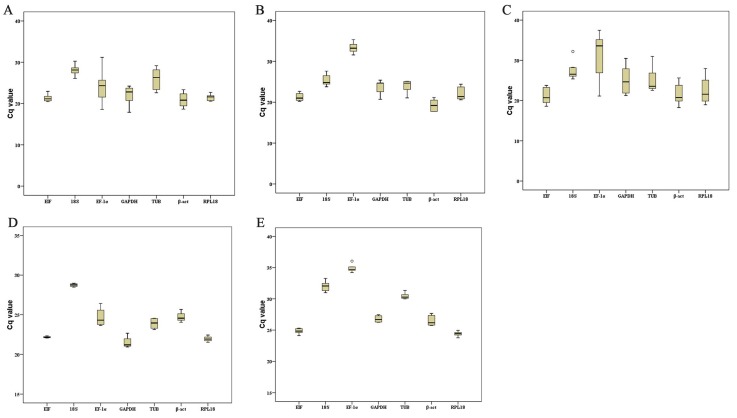
Variation in the reference genes expression using Cq values with boxplot. (**A**) Different tissues in adult shrimps. (**B**) Five different ovary development stages. (**C**) Different embryo development stages. (**D**) Different times after hypoxia stress in gill. (**E**) Different times after infection in hepatopancreas. Upper and lower whiskers mean maximum and minimum values, except outliers, and using a circle to mark the mild outliers and an asterisk to mark the extreme outliers. The upper and lower edges of the box represent the upper and lower quartiles, the middle black line is the median, and the whiskers represent the maximum and minimum values. Mild and extreme outliers are marked by circles and asterisks, respectively. The length of each graphic reflects its variation.

The quantitative expression levels of each reference gene under various tissues and conditions, according to their Cq value, were represented by line charts and boxplots. The line charts showed that the reference genes remained stable within each tissue under different conditions, while some genes varied widely among different tissues, such as *EF-1α* in adult and embryonic tissue (Figure 3). The overall distribution and data dispersion [28] are illustrated by boxplots. These plots indicated that *EIF*, *RPL18*, *β-act*, and *TUB* showed small discrete fluctuations, while *EF-1α* and *GAPDH* varied widely between the adult tissues and embryo development stages (Figure 4).

### 2.4. Stability Analysis 

We analyzed the stabilities of the reference genes using four commonly used methods, comparative ∆C_t_, BestKeeper, NormFinder, and GeNorm [20,21,22,23] (Table A2 and Table A3). Comparative ∆C_t_, BestKeeper, and NormFinder recommended RPL18 as the most stable reference gene in different adult tissues, while GeNorm identified that the combination of *EIF* and *RPL18* as the most stable genes. *TUB*, *GAPDH*, and *EF-1α* were the least stable genes according to all four methods. The comparative ∆C_t_ identified *β-act* as the most stable gene in the different ovarian stages, while BestKeeper and NormFinder considered *EIF* to be the best, and GeNorm considered the combination of *18S*/*RPL18* as the most stable target. *GAPDH* was the least stable gene according to all of these methods. Comparative ∆C_t_ and NormFinder considered *EIF* as the best reference gene in different embryonic stages, while BestKeeper picked *18S* and GeNorm chose the combination of *18S*/*TUB*. *GAPDH*, *RPL18*, and *EF-1α* were ranked the least stable. The comparative ∆C_t_ and NormFinder ranked *β-act* as the most stable reference gene in the *M. nipponense* gills under various durations of hypoxic stress, compared with *EIF* and *GAPDH*/*β-act*, according to GeNorm. *EF-1α* was not considered to be suitable in this situation. In the case of a *WSSV* infection, BestKeeper and NormFinder selected *RPL18*, comparatively ∆C_t_ picked *EIF*, and GeNorm selected *EIF*/*TUB* as the best reference genes, while all four ranked *β-act* and *18S* as the least stable.

**Figure 5:**
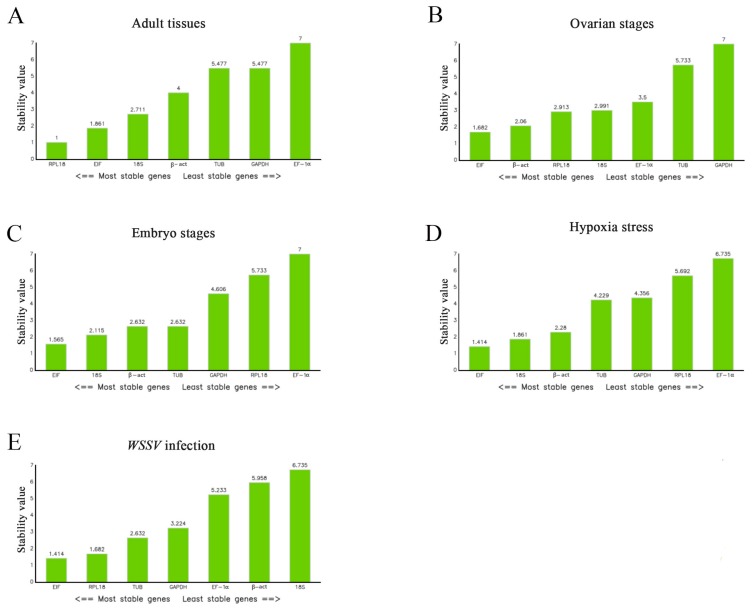
Comprehensive ranking of the reference genes under different conditions by RefFinder. (**A**) Different tissues in adult shrimps. (**B**) Five different ovary development stages. (**C**) Different embryo development stages. (**D**) Different times after hypoxia stress in gill. (**E**) Different times after infection in hepatopancreas. From left to right means the most suitable to the least suitable.

The comprehensive scores for these methods were ranked using RefFinder to give a total ranking (Figure 5) [23]. The genes ranked from most- to least-stable in the adult *M. nipponense* tissues were *RPL18*, *EIF*, *18S*, *β-act*, *TUB*, *GAPDH*, and *EF-1α*. The equivalent ranking in the different ovarian stages was *EIF*, *β-act*, *RPL18*, *18S*, *EF-1α*, *TUB*, and *GAPDH*, while that in the different embryonic stages was *EIF*, *18S*, *β-act*, *TUB*, *GAPDH*, *RPL18*, and *EF-1α*. The most- to least-stable genes under hypoxic stress were *β-act*, *GAPDH*, *EIF*, *18S*, *TUB*, *EF-1α*, and *RPL18*. *EIF* and *RPL18* were ranked highly under *WSSV* infection, followed by *TUB* and *GAPDH*, while *EF-1α*, *β-act*, and *18S* were not considered to be stable.

### 2.5. Validation of the Selected Reference Genes by RNAi

**Figure 6:**
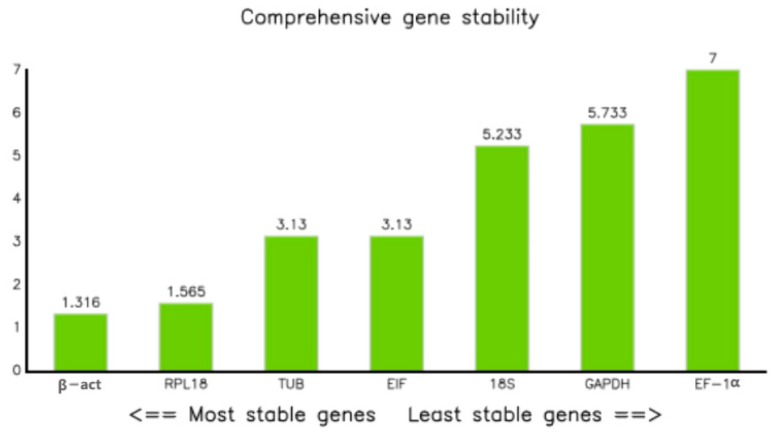
Comprehensive ranking of reference genes of RNAi.

**Figure 7:**
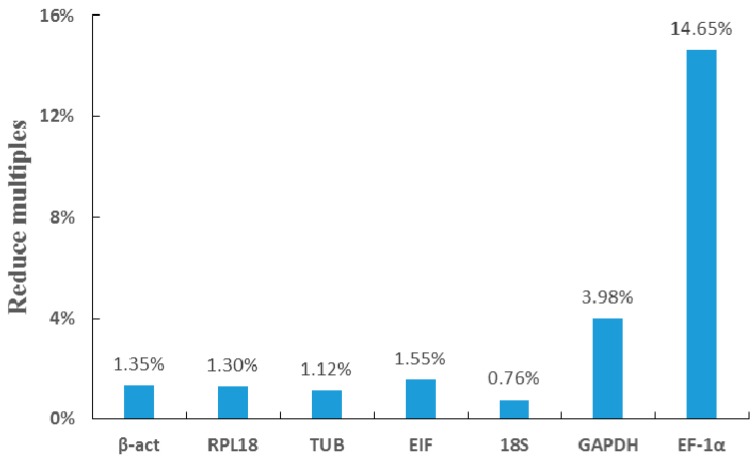
Using different genes as the reference gene for the reduce multiples of the *SST* gene expression, relative to the original level after interference.

We confirmed the selected reference genes and verified their rankings based on qPCR by RNAi, which could reliably decrease the expression level of the target gene in *M. nipponense* [29,30]. The *SST* expression in the androgenic glands in *M. nipponense* was predicted to decrease by >90% over seven days after RNAi. qPCR was performed to measure the expression difference in the expression levels based on different reference genes. The Cq values show that the difference in the tissue expression was not significant before and after the experiment, except *EF-1α* and *GAPDH* (Table 3). The three most stable reference genes were *β-act*, *RPL18*, and *TUB* (Table 4, Figure 6), and the expression of *SST*, measured using different reference genes, were reduced to 1.35%, 1.20%, and 1.12%, respectively (Figure 7), which confirms the success of the interference experiment. *β-act* was chosen as the best reference gene, which indicated a decrease in expression of *SST* to 1.35%. *EF-1α* was considered the least stable gene, and indicated a decrease in *SST* to 14.65% (Figure 7).

## 3. Discussion

Molecular studies in crustaceans have indicated that many genes tend to change their expression levels under different situations or in different stages of development [29,31]. qPCR is a useful tool for analyzing gene expression, with good specificity, accuracy, efficiency, and reproducibility. However, numerous studies have shown that the reference genes, such as *GAPDH* and *β-act*, that are used to normalize the data in qPCR studies may not remain stable under all conditions [10,32,33].

In this study, we screened out seven reference genes (*EIF*, *β-act*, *RPL18*, *18S*, *EF-1α*, *TUB*, and *GAPDH*), and ranked and evaluated them in *M. nipponense* under different situations. We initially chose candidate reference genes based on the recommended gene studied in the model organisms and other crustaceans. It is interesting that, although there are evolutionary and organizational differences in the different species, reference genes like *alpha-1,2-mannosyltransferase* in *Saccharomyces cerevisiae* [34], *heterogeneous nuclear ribonucleoprotein 27C* and *TBC1 domain family member 22A* in *Oryza sativa* [35], *60S ribosomal* protein *L13a* in *Danio rerio* [36], *TATA-box binding* protein, and *eukaryotic translation elongation factor 2* in *Mus musculus* [32] can be found in the mRNA transcriptomes studied in our lab. The genes were further selected after following inspection with gel electrophoresis and sequencing. For example, the *TATA-binding protein* was eliminated because the PCR product was too weak in the gel electrophoresis and it was not widely present in all organizations. These results indicated that some genes considered as classical reference genes in some species may not be suitable in other species. Previous studies of crustaceans, especially shrimps, identified *EIF* and *18S* as the most stable reference genes in different tissues in *Procambarus clarkii* [9], while *GAPDH* and *β-act* were chosen in different male tissues in *Macrobrachium rosenbergii* [15], and *EF-1α* in the reproductive system in the *Peneaus monodon* [13] and the infected experiment in *Penaeus stylirostris* [12]. However, *EF-1α* was almost the least stable in all situations, including in *WSSV*-infected experiment in *M. nipponense*, suggesting that reference genes are not necessarily interchangeable in different shrimp, even under the same conditions. *RPL18* has rarely been used as a reference gene in crustaceans, although it was selected as a reference gene in different developmental stages and tissues in *Solenopsis invicta* [37], and in the different developmental stages in *Anastrepha obliqua* [38]. *RPL18* performed well in seven adult tissues in the current study, but was the least stable gene in the different embryo stages, suggesting that some reference genes only performed well in specific tissues or conditions. These results demonstrate the need to screen for specific suitable reference genes before conducting qPCR studies under new experimental conditions or means of stress. The Cq values and stability rankings of *GAPDH* and *EF-1α* both varied widely under different experimental conditions, and it is therefore not recommended to use them as reference genes in qPCR of *M. nipponense*. In contrast, *EIF* performed well in most of the stability rankings and should therefore be considered as a conventional reference gene for qPCR studies in *M. nipponense.*

An experiment with significant differences was designed and performed to further confirm the reliability of the screening results. Previous researchers in our lab have proved that RNAi is able to significantly reduce the expression of specific genes in *M. nipponense* [29,30]. The *SST* gene is specifically expressed in androgenic glands [39], and its expression was predicted to decrease by >90% by seven days after the injection. We analyzed this decrease using each of the seven candidate reference genes and showed that *β-act* and *RPL18* were the most stable reference genes, with similar ratings, followed by *TUB* and *EIF* tied for third, and then *18S*, *GAPDH*, and *EF-1α*. According to the qPCR data, the expression of *SST* in the androgenic glands dropped to 1.3%, based on the two most stable reference genes, compared with a drop to 14.6% compared with the least stable gene, *EF-1α*. This result was in accord with the stability rankings. A previous study identified *EF-1α* as the most suitable reference gene for measuring moderate and highly expressed genes in the infected *P. stylirostris*, while *GAPDH* was a better control for the lower expressed genes, corresponding to the expressions of *EF-1α* and *GAPDH* [12]. The current study showed that *18S*, with *EIF*, nearly showed (0.58 and 0.44) a difference in the mean Cq values, and a much slighter difference than *GAPDH* (1.80), but the rank score much lower (5.233 and 3.13) and the qPCR data shows its decline difference (0.76% and 1.5%), which might indicate that, according to the expression level of the target gene, a medium high expression abundance of the reference gene was better. This suggests that some low-ranked genes may be suitable reference genes if the expression level of the target gene is very high or very low, depending on the Cq of the target gene. For example, *18S* may be suitable for target genes with low expression levels in adult tissues and under hypoxic stress, and *β-act* may be for highly expressed target genes in different ovarian stages in *M. nipponese* (Figure 3, Figure 4 and Figure 5).

In this study, we screened seven potential reference genes’ potential for *M. nipponense* from a large number of candidate genes, and analyzed their stability under various tissues and under different stresses using qPCR. *EIF* was stable in different situations, especially in *WSSV* infection and in different ovarian and embryo stages, while *RPL18* was the most stable reference gene in the adult tissues, and *β-act* was the best reference gene under hypoxia stress and RNAi. The five tools (GeNorm, NormFinder, BestKeeper, the comparative ∆C_t_ method, and RefFinder) used in this research for analyzing the stability rankings of the reference gene were widely used in crustaceans [12,13,14,15,16], but the reference genes have rarely been screened for conditions of hypoxia and RNAi. The current reference genes, identified under different conditions in *M. nipponense*, will thus provide a useful references for qPCR experiments in other crustaceans.

## 4. Materials and Methods 

### 4.1. Selection of Reference Genes and Primer Design

The candidate gene selection was chosen from the reference genes that had been studied in other model animals and similar species. Then, their names were used to search the existing transcriptome libraries and the genes that were extensive in organizations were selected. The sequences were obtained from the transcriptome library. Nucleotide sequences were analyzed based on the nucleotide and protein databases using the BLASTX and BLASTN programs (http://www.ncbi.nlm.nih.gov/BLAST/). The protein prediction was performed using the open reading frame (ORF) finder (http://www.ncbi.nlm.nih.gov/gorf/). Multiple alignments of amino acid sequences’ encoding were created using DNAMAN 6.0 [30].

All of the primers for the experiments were designed based on the open reading frame and using Primer-Blast tools in NCBI (http://www.ncbi.nlm.nih.gov/tools/primer-blast/). A reaction without RNA templates was used as the negative control. The primers were designed to verify the ORFs of those sequences. After getting reliable ORFs, the primers for qPCR were designed considering the following parameters: primer size, 22–24 bp; product size, 150–300 bp; annealing temperature, 59–61 (°C) and a GC(composition) content of 40–60%. In order to get the most suitable primers, three attempts were taken to design and each time, with four pairs of primers design per gene.

### 4.2. Amplification Efficiency and Primers Specificity of Reference Genes

The specificity must be validated empirically with direct experimental evidence (electrophoresis gel, melting profile, DNA sequencing, amplicon size, and/or restriction enzyme digestion) [3]. The melting curve was measured to evaluate the specificity of the reference genes. The efficiency was based on the slope of a linear regression model and was calculated from the slope of a standard curve. The efficiencies (E) and correlation coefficients (R^2^) were calculated for each reference gene [40]. The PCR efficiencies of the primer pairs were determined by qPCR, using a serial dilution of pooled hepatopancreas cDNA samples (×1, ×10, ×100, ×1000, and ×10000 dilutions).

### 4.3. Animals, Stress Experiments, and Tissue Collection 

Our study does not involve endangered or protected species. This study was approved by the Institutional Animal Care and Use Ethics Committee of the Freshwater Fisheries Research Center, the Chinese Academy of Fishery Sciences (Wuxi, China, FFRC125, 26 August 2016). All of the prawns were obtained from the Freshwater Fisheries Research Center, the Chinese Academy of Fishery Sciences, Wuxi, China (120°13′44″ E, 31°28′22″ N) [30].

In the spatiotemporal expression study, after one week, the laboratory culture, ovary, testis, gill, muscle, eyestalk, hepatopancreas, and heart were dissected out of the mature prawns (*n* = 3). The developmental stage of the embryo was classified into seven stages based on the criteria of Qiao et al. (2015) [41]. The ovarian cycle of the prawns was classified into five stages based on the previous results Gao et al., (2006) [42].

For the hypoxia stress experiment, every 30 shrimp in three tanks were maintained within the treatment tanks for 0, 3, 6, 12, and 24 h by nitrogen-filled manipulation in the hypoxic conditions (2.0 ± 0.2 mg O_2_ L^−1^). The hypoxic dissolved oxygen value was set on the basis of the previous observations of juvenile *M. nipponense* [43]. A gill of five time points of these shrimp was collected and all of the exposures were conducted in triplicate.

In the white spot syndrome virus (*WSSV*) infection, every 20 healthy shrimp in three tanks were bred for seven days in a laboratory condition, before infection. All of the experimental materials and methods are referenced from Zhao et al. (2017) [44]. The injection positions were between the third and fourth abdominal segments. Hepatopancreas was collected in triplicate at 0, 12, 24, 48, 72, and 96 h after post-inoculation (hpi) with *WSSV*.

### 4.4. RNA Extraction and cDNA Synthesis 

All of the samples mentioned above were dissected out and frozen immediately in liquid nitrogen, and stored at −80 °C until processed. The total RNA was isolated from the different tissues of the prawns using RNAiso Plus Reagent (TaKaRa, Kusatsu, Japan), according to the manufacturer’s protocols. At least three shrimp were analyzed for each type of tissue. For all of the RNA samples, the A260/A280 ratios were in the range of 1.9–2.1, and the RNA integrity was verified by 1.2% agarose gel electrophoresis and stored at 80 °C.

Approximately 1 μg of the total RNA from each tissue was reverse-transcribed by the iScript™ cDNA Synthesis Kit Perfect Real Time (BIO-RAD, Hercules, CA, USA), according to the manufacturerr’s instructions. Synthesized cDNA were diluted to 2-fold and stored at −20 °C until use.

### 4.5. Quantitative Real-Time PCR (qPCR)

The qPCR amplification was performed in a total volume of 25 μL, which contained 1 μL cDNA (50 ng), 10 μL SsoFast™ EvaGreen Supermix (BIO-RAD, Hercules, CA, USA), 0.5 μL (10 μM) of the primers (Table 1), and 13 μL of nuclease-free water. The reaction mixture was initially incubated at 95 °C for 30 s to activate the Hot Start Taq DNA polymerase, followed by 40 cycles at 95 °C for 10 s and 60 °C for 10 s, and a melting cure analysis was performed at the end of the qPCR reaction at 65–95 °C (with increments of 0.5 °C) for 10 s. The differences of the expressions turned out to be significant (*p* < 0.05).

### 4.6. Methods for Analyzing Stability of Reference Genes

The expression stabilities of the candidate reference genes for the tissues and embryos were analyzed separately using RefFinder, an algorithm that integrates four widely used computational programmes for the analysis of the expression stability, namely GeNorm, NormFinder, BestKeeper, and the comparative ∆C_t_ method. Briefly, based on the rankings provided by individual programmes, RefFinder assigns an appropriate weight to each gene and calculates the geometric mean of the weights, in order to give an overall ranking [23].

### 4.7. Validation of Reference Genes by SST Gene RNA Interference 

For the in vivo dsRNA injection experiment, 50 healthy mature male prawns (weights of 2.4 ± 0.6 g) were assigned to two groups. The experimental group (*N* = 25) was injected with *SST* dsRNA. Each prawn was injected with *SST* dsRNA through the pericardial cavity membrane of the carapace at a dose of 4 μg/g.b.w. The control group (*N* = 25) was injected with diethy pyrocarbonate water at volumes equivalent to those applied to the experimental group (based on gram body weight). After the injection, 10 prawns from each group were randomly collected on the seventh day. The primer for the dsRNA of the *SST*, named *dsSST*, was designed using Snap Dragon tools (available online: http://www.flyrnai.org/cgi-bin/RNAi_find_primers.pl), and it is displayed in Table A1. The primers used for the qPCR of the *SST* was referred to in Jin et al. (2014) [39]. The purity and integrity of the double-stranded RNA (dsRNA) followed the instructions of the Transcript AidTMT7 High Yield Transcription kit (Fermentas, Waltham, USA).

## Figures and Tables

**Table 1:** Primer pairs for qPCR of candidate reference genes. EIF—eukaryotic translation initiation factor 5A; 18S—18S ribosomal RNA; EF-1α—elongation factor-1α; GAPDH—glyceraldehyde-3-phosphate dehydrogenase; TUB—α-tubulin; β-act—β-actin; RPL18—Ribosomal protein L18.

Gene	Primer Sequence (5′–3′) Forward/Reverse	Length (bp)	PCR Efficiency (%)	Correlation Coefficient (R^2^)
*EIF*	CATGGATGTACCTGTGGTGAAAC CTGTCAGCAGAAGGTCCTCATTA	179	94.4	0.994
*18S*	GTTGGATGTTGCTGTTGAGAGAG CTGGGCATCATTCTCTGGGTAAA	250	96.8	0.997
*EF-1α*	CAAGGATCTGAAACGTGGCTTC GTACGTCTGTCGATCTTGGTCAG	198	93.7	0.999
*GAPDH*	GTCGGTAAGGTCATTCCAGAGC CGAAAGTTTTGCTGAGCTGGAT	274	95.5	0.991
*TUB*	AGGAATGGAAAATCAGGAAGCCC GTTTGTCGATCTGGAACCCTCT	224	97.0	0.996
*β-act*	CACGAGACCACCTACAATTCCA ATAGAGAAGCCAAGATAGAACCGC	226	99.4	0.991
*RPL18*	CTTTTTGTACCCACAGCTTGACC CACTTTTGATGTATTGGCCCGTC	202	102.7	0.997

**Table 2:** Detailed distribution of cycle threshold (Cq) value information.

Experimental Conditions	Statistics	Reference Gene						
		*EIF*	*18S*	*EF* *-* *1* *α*	*GAPDH*	*TUB*	*β-act*	*RPL18*
Hypoxia stress	Group	5	5	5	5	5	5	5
	Mean	21.86	28.19	24.20	21.19	23.80	24.35	21.33
	Min Cq	20.64	26.08	22.50	20.02	23.13	23.01	19.03
	Max Cq	22.33	28.98	26.42	22.66	24.56	25.66	22.43
*WSSV* infection	Group	6	6	6	6	6	6	6
	Mean	24.82	32.02	34.89	26.78	30.44	26.47	24.40
	Min Cq	24.12	30.97	34.22	26.26	29.98	25.72	23.76
	Max Cq	25.30	33.28	36.04	27.44	31.34	27.64	24.96
Different adult tissues	Group	7	7	7	7	7	7	7
	Mean	21.65	27.81	25.05	22.55	25.86	20.82	21.83
	Min Cq	20.47	26.09	18.57	17.90	22.61	18.64	20.56
	Max Cq	23.59	30.26	32.62	26.74	29.17	23.36	23.88
Different ovarian stages	Group	5	5	5	5	5	5	5
	Mean	21.29	25.42	33.34	23.61	23.81	19.28	22.18
	Min Cq	20.24	23.77	31.56	20.71	21.06	17.75	20.59
	Max Cq	22.66	27.62	35.32	25.40	25.11	21.19	24.40
Different embryo stages	Group	7	7	7	7	7	7	7
	Mean	21.23	27.51	30.90	25.13	25.25	21.71	22.63
	Min Cq	18.58	25.37	21.12	21.25	22.53	18.26	18.95
	Max Cq	23.77	32.10	37.48	30.47	30.98	25.62	27.97

‘Group’ in this table represents how many states or tissues the genes are studied in. Each means five time points during hypoxia stress, six time points during *WSSV* infection, seven different organizations in adult tissues study, five different ovarian stages, and seven different embryo stage. *WSSV*—white spot syndrome virus.

**Table 3:** Detailed Cq value sorting of RNAi.

Experimental Conditions	Statistics	Reference Gene						
		*EIF*	*18S*	*EF* *-* *1* *α*	*GAPDH*	*TUB*	*β-act*	*RPL18*
RNAi (Androgenic glands)	Group	2	2	2	2	2	2	2
	Mean	21.29	27.62	24.51	22.14	23.46	19.11	20.58
	Min Cq	21.07	27.33	22.63	21.34	23.40	18.99	20.55
	Max Cq	21.51	27.91	26.38	23.14	23.51	19.23	20.62

‘Group 2’ in this table represents two states, before and after the RNAi experiment.

**Table 4:** Ranking of reference genes of RNAi by different methods.

Experimental Conditions	Method	Rank						
		1	2	3	4	5	6	7
RNAi (Androgenic glands)	Comparative ∆C_t_	*β-act*	*RPL18*	*EIF*	*TUB*	*18S*	*GAPDH*	*EF-1α*
	BestKeeper	*RPL18*	*TUB*	*β-act*	*EIF*	*18S*	*GAPDH*	*EF-1α*
	NormFinder	*β-act*	*EIF*	*RPL18*	*TUB*	*GAPDH*	*18S*	*EF-1α*
	GeNorm	*β-act/RPL18*		*TUB*	*EIF*	*18S*	*GAPDH*	*EF-1α*
	Recommended comprehensive ranking	*β-act*	*RPL18*	*TUB*	*EIF*	*18S*	*GAPDH*	*EF-1α*

## Abbreviations

qPCR	quantitative real-time reverse transcription PCR
ORF	open reading frame
RNAi	RNA interference
*M. nipponense*	*Macrobrachium nipponense*

## Appendix A

**Table A1:** Primers for validating open reading frames.

Abbreviation	Gene Name	Primer Sequence (5′–3′) Forward/Reverse	Genbank Accession No.
*EIF*	*eukaryotic translation initiation factor 5A*	GTTGTATGCAGTCGGCCATATTT TGTCCTGAAGGTGGTGATAATGA	MH540106
*18S*	*18S ribosomal RNA*	CTCCCCCTGAAGTGTATTATGGA GCGAATCTTTTTCAGTTGTTCCC	MH540107
*EF-1α*	*elongation factor 1-alpha*	CCTGGTATGGTTGTCACTTTTGC CTCCTTCTTCGACACCTCTTTGA	MH540108
*GAPDH*	*glyceraldehyde-3-phosphate dehydrogenase*	CCAAGGACATGAAGGTAGTCTCC TTCTGCATGTGCTTCAACAAGTC	MH540109
*TUB*	*α-tubulin*	GGTCTGGAATTCAGTCAAGTCG AGTGCATCTCAGTTCATGTTGG	MH540110
*β-act*	*β-actin*	CTCTTCTTCCCTGGAGAAGTCTTA ATCCACATCTGTTGGAAGGTAGA	MH540111
*RPL18*	*ribosomal protein L18*	ACTTTTTGTACCCACAGCTTGAC GTGAAGGGCGAATCTTTGTTGTT	MH540112
*dsSST*	*slow-tonic S2 tropomyosin*	TAATACGACTCACTATAGGGCGAGAGGTCTGAGGAACGAC TAATACGACTCACTATAGGGTAAGCTTCCTCACGCTGGTT	

**Table A2:** Ranking of reference genes under different conditions by different methods.

Experimental Conditions	Method	Rank						
		1	2	3	4	5	6	7
Different adult tissues	Comparative ∆C_t_	*RPL18*	*EIF*	*β-act*	*18S*	*TUB*	*GAPDH*	*EF-1α*
	BestKeeper	*RPL18*	*EIF*	*18S*	*β-act*	*TUB*	*GAPDH*	*EF-1α*
	NormFinder	*RPL18*	*EIF*	*β-act*	*18S*	*GAPDH*	*TUB*	*EF-1α*
	GeNorm	*EIF/RPL18*		*18S*	*β-act*	*TUB*	*GAPDH*	*EF-1α*
	Recommended comprehensive ranking	*RPL18*	*EIF*	*18S*	*β-act*	*TUB*	*GAPDH*	*EF-1α*
Different ovarian stages	Comparative ∆C_t_	*β-act*	*EIF*	*RPL18*	*18S*	*EF-1α*	*TUB*	*GAPDH*
	BestKeeper	*EIF*	*EF-1α*	*β-act*	*18S*	*TUB*	*RPL18*	*GAPDH*
	NormFinder	*EIF*	*β-act*	*EF-1α*	*RPL18*	*18S*	*TUB*	*GAPDH*
	GeNorm	*18S/RPL18*		*β-act*	*EIF*	*EF-1α*	*TUB*	*GAPDH*
	Recommended comprehensive ranking	*EIF*	*β-act*	*RPL18*	*18S*	*EF-1α*	*TUB*	*GAPDH*
Different embryo stages	Comparative ∆C_t_	*EIF*	*β-act*	*TUB*	*18S*	*GAPDH*	*RPL18*	*EF-1α*
	BestKeeper	*18S*	*EIF*	*β-act*	*TUB*	*RPL18*	*GAPDH*	*EF-1α*
	NormFinder	*EIF*	*β-act*	*GAPDH*	*TUB*	*18S*	*RPL18*	*EF-1α*
	GeNorm	*18S/TUB*		*EIF*	*β-act*	*GAPDH*	*RPL18*	*EF-1α*
	Recommended comprehensive ranking	*EIF*	*18S*	*β-act*	*TUB*	*GAPDH*	*RPL18*	*EF-1α*
Hypoxia stress	Comparative ∆C_t_	*β-act*	*GAPDH*	*EIF*	*18S*	*EF-1α*	*TUB*	*RPL18*
	BestKeeper	*EIF*	*TUB*	*GAPDH*	*β-act*	*18S*	*RPL18*	*EF-1α*
	NormFinder	*β-act*	*GAPDH*	*EIF*	*18S*	*TUB*	*EF-1α*	*RPL18*
	GeNorm	*GAPDH* */* *β-act*		*EIF*	*18S*	*EF-1α*	*TUB*	*RPL18*
	Recommended comprehensive ranking	*β-act*	*GAPDH*	*EIF*	*18S*	*TUB*	*EF-1α*	*RPL18*
*WSSV* infection	Comparative ∆C_t_	*EIF*	*RPL18*	*GAPDH*	*TUB*	*EF-1α*	*β-act*	*18S*
	BestKeeper	*RPL18*	*EIF*	*TUB*	*GAPDH*	*EF-1α*	*18S*	*β-act*
	NormFinder	*RPL18*	*EIF*	*GAPDH*	*TUB*	*β-act*	*EF-1α*	*18S*
	GeNorm	*EIF /TUB*		*GAPDH*	*RPL18*	*EF-1α*	*β-act*	*18S*
	Recommended comprehensive ranking	*EIF*	*RPL18*	*TUB*	*GAPDH*	*EF-1α*	*β-act*	*18S*

**Table A3:** Reference gene expression stability values based on several programs.

	Rank	∆Ct		BestKeeper		NormFinder		GeNorm	
		Genes	Average of Std dev	Genes	Std dev	Genes	Stability Index	Genes	Stability Value
Different adult tissues	1	*RPL18*	2.22	*RPL18*	0.83	*RPL18*	0.252	*EIF/RPL18*	0.392
	2	*EIF*	2.24	*EIF*	0.94	*EIF*	0.542		
	3	*β-act*	2.52	*18S*	1.10	*β-act*	1.767	*18S*	1.354
	4	*18S*	2.66	*β-act*	1.38	*18S*	1.934	*β-act*	1.55
	5	*TUB*	2.91	*TUB*	1.93	*GAPDH*	2.128	*TUB*	1.705
	6	*GAPDH*	3.07	*GAPDH*	2.16	*TUB*	2.369	*GAPDH*	2.192
	7	*EF-1α*	4.65	*EF-1α*	3.74	*EF-1α*	4.462	*EF-1α*	2.895
Different ovarian stages	1	*β-act*	0.85	*EIF*	0.87	*EIF*	0.439	*18S/RPL18*	0.319
	2	*EIF*	0.88	*EF-1α*	1.11	*β-act*	0.506		
	3	*RPL18*	0.91	*β-act*	1.25	*EF-1α*	0.609	*β-act*	0.388
	4	*18S*	0.95	*18S*	1.3	*RPL18*	0.637	*EIF*	0.513
	5	*EF-1α*	0.99	*TUB*	1.38	*18S*	0.749	*EF-1α*	0.653
	6	*TUB*	1.2	*RPL18*	1.5	*TUB*	1.032	*TUB*	0.892
	7	*GAPDH*	1.33	*GAPDH*	1.57	*GAPDH*	1.218	*GAPDH*	1.016
Different embryo stages	1	*EIF*	2.37	*18S*	1.89	*EIF*	0.642	*18S/TUB*	1.165
	2	*β-act*	2.47	*EIF*	1.73	*β-act*	0.786		
	3	*TUB*	2.74	*β-act*	5.07	*GAPDH*	1.775	*EIF*	1.44
	4	*18S*	2.77	*TUB*	3.11	*TUB*	1.906	*β-act*	1.578
	5	*GAPDH*	2.84	*RPL18*	2.55	*18S*	2.059	*GAPDH*	1.724
	6	*RPL18*	3.36	*GAPDH*	2.3	*RPL18*	2.295	*RPL18*	2.24
	7	*EF-1α*	5.35	*EF-1α*	2.92	*EF-1α*	5.137	*EF-1α*	3.129
Hypoxia stress	1	*β-act*	0.6	*EIF*	0.49	*β-act*	0.092	*GAPDH*/*β-act*	0.185
	2	*GAPDH*	0.66	*TUB*	0.57	*GAPDH*	0.316		
	3	*EIF*	0.7	*GAPDH*	0.61	*EIF*	0.323	*EIF*	0.434
	4	*18S*	0.74	*β-act*	0.67	*18S*	0.45	*18S*	0.532
	5	*EF-1α*	0.95	*18S*	0.85	*TUB*	0.822	*EF-1α*	0.631
	6	*TUB*	0.97	*RPL18*	0.93	*EF-1α*	0.828	*TUB*	0.72
	7	*RPL18*	1.01	*EF-1α*	1.06	*RPL18*	0.912	*RPL18*	0.803
*WSSV* infection	1	*EIF*	0.66	*RPL18*	0.31	*RPL18*	0.297	*EIF*/*TUB*	0.397
	2	*RPL18*	0.67	*EIF*	0.32	*EIF*	0.334		
	3	*GAPDH*	0.69	*TUB*	0.38	*GAPDH*	0.391	*GAPDH*	0.454
	4	*TUB*	0.72	*GAPDH*	0.41	*TUB*	0.424	*RPL18*	0.489
	5	*EF-1α*	0.91	EF-1α	0.46	*β-act*	0.737	*EF-1α*	0.593
	6	*β-act*	0.92	18S	0.6	*EF-1α*	0.748	*β-act*	0.703
	7	*18S*	1.06	β-act	0.68	*18S*	0.929	*18S*	0.805
